# Protective Effects of Berberine on Renal Injury in Streptozotocin (STZ)-Induced Diabetic Mice

**DOI:** 10.3390/ijms17081327

**Published:** 2016-08-12

**Authors:** Xiuli Zhang, Hui He, Dan Liang, Yan Jiang, Wei Liang, Zhi-Hong Chi, Jianfei Ma

**Affiliations:** 1 Department of Nephrology, Benxi Center Hospital, 29 Victory Road, Benxi 117000, Liaoning, China; 2 Science Experiment Center, Benxi Center Hospital, Benxi 117000, Liaoning, China; lnbxwxf@yeah.net (H.H.); liangbixiang@sohu.com (W.L.); 3 Key Laboratory of Medical Cell Biology of Ministry of Education, China Medical University, Shenyang 110001, Liaoning, China; 4 Troops of 95935 Unit, Haerbin 150111, Heilongjiang, China; dl7711@163.com; 5 Department of pathophysiology, China Medical University, Shenyang 110001, Liaoning, China; chizhihong2015@126.com; 6 Department of Nephrology, the First Affiliated Hospital, China Medical University, Shenyang 110001, Liaoning, China; jfeima@sohu.com

**Keywords:** renal tubular epithelial cells, berberbine, EMT, diabetic nephropathy, Nrf2 pathway, TGF-β/Smad signaling pathway

## Abstract

Diabetic nephropathy (DN) is a serious diabetic complication with renal hypertrophy and expansion of extracellular matrices in renal fibrosis. Epithelial-to-mesenchymal transition (EMT) of renal tubular epithelial cells may be involved in the main mechanism. Berberine (BBR) has been shown to have antifibrotic effects in liver, kidney and lung. However, the mechanism of cytoprotective effects of BBR in DN is still unclear. In this study, we investigated the curative effects of BBR on tubulointerstitial fibrosis in streptozotocin (STZ)-induced diabetic mice and the high glucose (HG)-induced EMT in NRK 52E cells. We found that BBR treatment attenuated renal fibrosis by activating the nuclear factor-erythroid 2-related factor 2 (Nrf2) signaling pathway in the diabetic kidneys. Further revealed that BBR abrogated HG-induced EMT and oxidative stress in relation not only with the activation of Nrf2 and two Nrf2-targeted antioxidative genes (NQO-1 and HO-1), but also with the suppressing the activation of TGF-β/Smad signaling pathway. Importantly, knockdown Nrf2 with siRNA not only abolished the BBR-induced expression of HO-1 and NQO-1 but also removed the inhibitory effect of BBR on HG-induced activation of TGF-β/Smad signaling as well as the anti-fibrosis effects. The data from present study suggest that BBR can ameliorate tubulointerstitial fibrosis in DN by activating Nrf2 pathway and inhibiting TGF-β/Smad/EMT signaling activity.

## 1. Introduction

Diabetic nephropathy (DN) is the leading cause of chronic kidney failure and end-stage renal disease [1,2]. In the early stage of DN, the patients exhibit with the increased kidney size, glomerular volume and kidney function, along with the accumulation of glomerular extracellular matrix and the increase of urinary albumin excretion, glomerular sclerosis and tubular fibrosis [2]. Moreover, once the patients with DN develop fibrosis in glomerulus and tubulointerstitium, their kidney structure and filtration are susceptible to be damaged [3,4]. Accumulating evidence has demonstrated that tubulointerstitial fibrosis usually develops at the early stage of diabetic renal injury and is also related to the decline of renal function, which happened to some diabetes patients [5,6]. In addition, hyperglycemia, advanced products of nonenzymatic protein glycation and an excess accumulation of extracellular matrix (ECM) are connected with prolonged injury, these factors also enhance the kidney fibrosis process [7,8]. To date, some studies has verified that EMT of mature tubular epithelial cells plays a crucial role in accelerating the progression of DN relative matrix proteins as well [9]. Thus, investigation of antifibrotic mechanisms remains a promising therapeutic target in the diabetic renal diseases [10].

Several studies have revealed that oxidative stress may be involved to the EMT of tubular epithelia cells in diabetes [11,12]. NF-E2-related factor 2 (Nrf2) is one of important cellular defense factors to counteract oxidative stress and regulates intracellular antioxidants and phase II detoxifying enzymes, which can detoxify xenobiotics and neutralize ROS so that promoting cell survival and maintaining cellular redox homeostasis [13,14]. In normal condition, Nrf2 is held as an inactive complex in the cytoplasm by the repressor molecule, Keap1 (Kelch-like ECH-associated protein 1), which can facilitate its ubiquitination once exposing to oxidative stress and/or electrophiles. When exposing to oxidative stress or electrophiles, Nrf2 occurs ubiquitination and translocates into nuclei and then activates the transcription of antioxidant genes such as NADPH quinone oxidoreductase-1 [NQO-1] and heme oxygenase-1 [HO-1]. Both genes can induce the production of anti-inflammatory, anti-fibrosis and anti-apoptotic metabolites [15,16,17]. In vivo studies also identificated that renal ischemia-reperfusion can elevate Nrf2 levels and activate their downstream targets in kidneys [18]. Meanwhile, targeting Nrf2 can not only mitigate cisplatin-induced nephrotoxicity but also attenuate cyclosporin A-induced EMT in renal fibrosis [19,20,21]. Additionally, there was a report showed that improving Nrf2 expression by sulforaphane can suppress ROS triggered by hyperglycemia and restored metabolic dysfunction in human microvascular endothelial cells [22]. In contrast, transcription factor Nrf2 deficiency promotes the deteripration of ischemic and nephrotoxic acute kidney injury [23]. Nrf2 knockout mice with STZ-induced diabetes increased their urinary nitric oxide metabolites levels (an indirect evidence of oxidative stress) and induced renal injury [14]. Thus, Nrf2 has been considered as a potential therapeutic target for renal diseases.

Berberine (BBR, C20H18NO4) is an isoquinoline alkaloid isolated from Coptidis Rhizoma and Cortex Phellodendri with multiple pharmacological activities [24,25]. BBR has a variety of biological effects, for instance, antioxidant, anti-inflammatory, anti-tumor and anti-fibrosis effects [25,26,27]. In addition, recent studies proofed that BBR ameliorated renal dysfunction in diabetic rodents and suppressed high glucose-induced glomerular mesangial cell proliferation by supperessing TGF-β1 expression and ECM accumulation, this function suggested BBR can as a potential drug for DN [28,29,30,31]. Furthermore, the antioxidant effect of BBR have been demonstrated it not only can improve renal function in certain diabetic animal models [32], but also ameliorate in endothelial function by reducing endothelial microparticles-mediated oxidative stress [33]. Most importantly, Yu et al. have recently proofed that BBR protects human renal proximal tubular cells from hypoxia/reoxygenation injury via inhibiting endoplasmic reticulum and mitochondrial stress [26]. However, the underlying molecular mechanisms of protection of BBR on tubulointerstitial fibrosis in DN remain unclear. Therefore, in the study, we investigate protective effects of BBR against renal injury in streptozotocin (STZ)-induced diabetic mice and the underlying mechanisms.

## 2. Results

### 2.1. The Effects of BBR on Metabolic and Biochemical Parameters in STZ-Induced Diabetic Mice

As showed in Table 1, STZ-induced diabetic mice exhibited typical diabetic symptoms compared with those in normal mice, for instance, increase in diet, drink and urine while weight loss. Fasting blood glucose (FBG) and kidney weight/body weight ratio (KW/BW) in diabetic mice were significantly higher but their body weight was loss. At the end of the 12th week, all blood serum creatinine (Cr), urea nitrogen (BUN) and 24-albuminuria were increased in diabetic mice. These results suggested that diabetic renal dysfunction was emerged (Table 1). The level of FBG, KW/BW, Cr, BUN and diabetic symptom were dramatically ameliorated in BBR-treatment mice compared with those in diabetic mice (Table 1). All these findings indicated that BBR could effectively cure the renal function in diabetic mice.

### 2.2. BBR Can Supress the Fibrosis in Diabetic Kidneys by Inhibiting EMT

**Figure 1:**
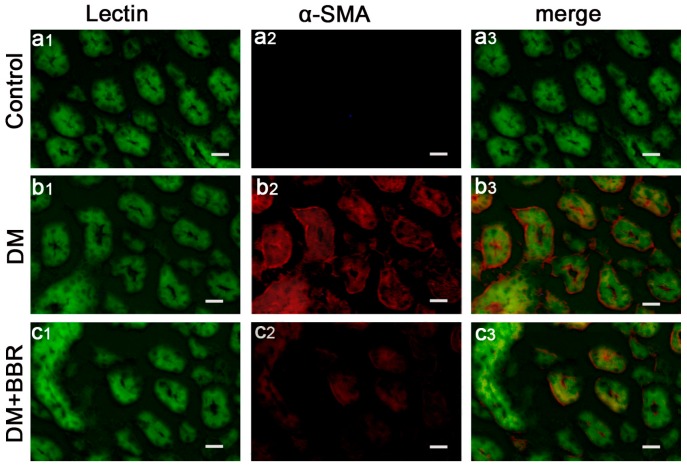
Double immunofluorescence staining shows localization of α-SMA (red) and the tubular cell marker lectin (green) in control (**a1**–**3**) and diabetic kidneys (**b1**–**3**); BBR-treated diabetic kidneys (**c1**–**3**) (respectively. Scale bar, 20 μm).

**Figure 2:**
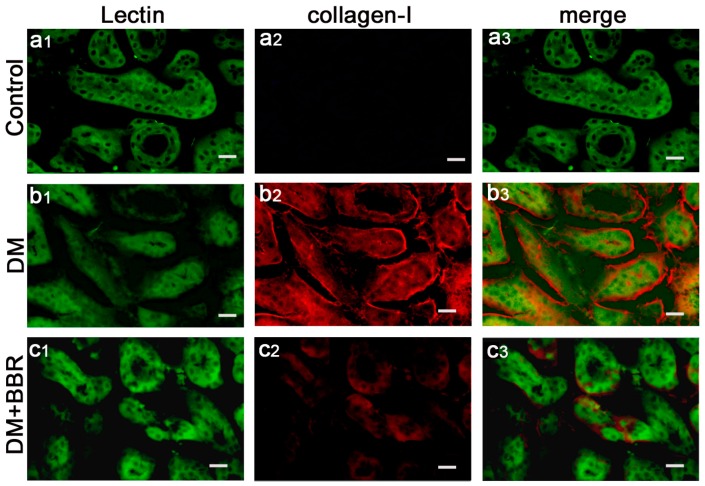
Double immunofluorescence staining shows localization of collagen-I (red) and the tubular cell marker lectin (green) in control (**a1**–**3**) and diabetic kidneys (**b1**–**3**); BBR-treated diabetic kidneys (**c1**–**3**) (respectively. Scale bar, 20 μm).

**Figure 3:**
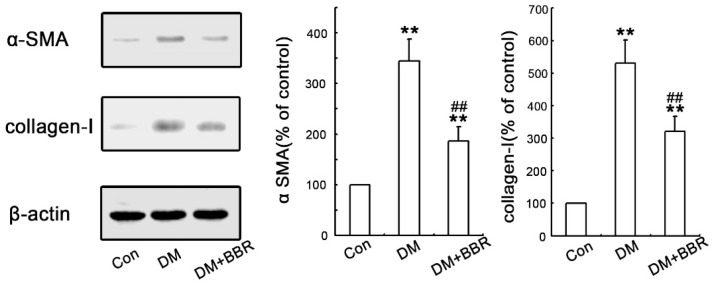
Antifibrotic effects of BBR were associated with the inhibition of EMT in diabetic kidneys. Representative western blots of α-SMA and collagen-I in the kidneys of control mice, diabetic mice or BBR-treated diabetic mice. All experiments were performed three times with virtually identical results. β-actin was used as loading control. (** *p* < 0.01 vs. control group; ## *p* < 0.01 vs. control diabetic group. *n* = 8).

In order to investigate the anti-fibrotic effects of BBR in connection with the inhibition of EMT in diabetic kidneys, double-immunofluorescence staining for Lectin (a tubular marker) and α-SMA (a mesenchymal marker) or collagen-I were used to analyze their expression and localization in the renal interstitium. The data showed that α-SMA protein (red) was specifically localized in the interstitial compartment and the tubular epithelial cells of diabetic kidneys, whereas α-SMA was virtually absent in sham control kidneys (Figure 1a1–3), these findings suggesting de α-SMA, as a special diagnostic markers, can be detected in in tubular epithelia expression under diabetic conditions (Figure 1b1–3). However, the staining of α-SMA of interstitial compartment and the tubular epithelial cells in the BBR-treated group was weaker than that in untreated diabetic mice (Figure 1c1–3). Additionally, double-immunofluorescence staining for Lectin and collagen-I was performed to detect the distribution of collagen-I protein in the renal interstitium. As shown in Figure 2, the expression of Collagen-I increased in tubularinterstitial apparatus in DN and BBR attenuated this response. Furthermore, Western blot also showed the BBR-treated groups significantly suppress the expression of α-SMA and collagen-I compared with those in diabetic kidneys (Figure 3). These observations were consistent with the results of immunofluorescence staining. It is clear that BBR decreased tubulointerstitial fibrosis in STZ-induced diabetic mice with nephropathy.

### 2.3. BBR Enhanced Activation of Nrf2/HO-1 Signaling in Diabetic Mice Kidneys

**Figure 4:**
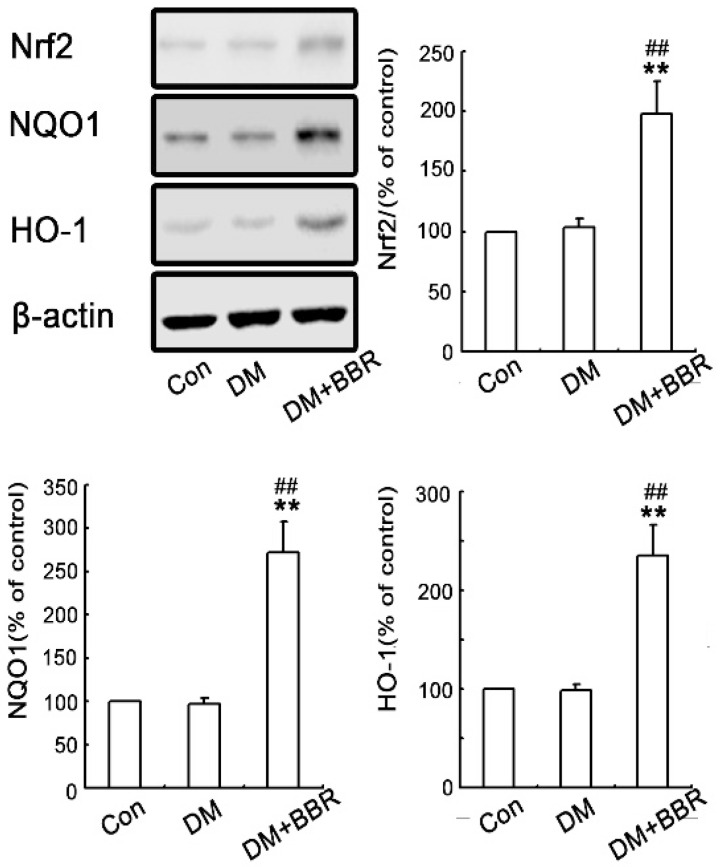
BBR enhanced activation of Nrf2/HO-1 signaling in diabetic kidneys. Representative western blots demonstrated that BBR treatment increased protein expression of Nrf2, NQO1 and HO-1 in the kidneys of diabetic mice (*n* = 8). Each value represents mean ± SEM. All experiments were performed three times with virtually identical results. β-actin was used as loading control. (** *p* < 0.01 vs. control group; ## *p* < 0.01 vs. control diabetic group. *n* = 8).

Oxidative stress plays important roles in initiating diabetic fibrotic effects. To further explore if the anti-fibrosis role of BBR is relate to Nrf2 signaling pathway, the expressions of Nrf2 relative genes (NQO1 and HO-1) in diabetic mice kidneys were detected. As shown in Figure 4, the renal Nrf2 expression slightly increased in diabetic group compared to those in control (*p* > 0.05) while BBR treatment can induce renal Nrf2 expression in diabetes group (Figure 4). Similarly, the expression of the Nrf2 target gene products including NQO1 and HO-1 were both found (Figure 4). These results indicated that BBR activated Nrf2-mediated anti-oxidative cascades in this model.

### 2.4. The Influence of BBR on HG-Induced EMT Markers in NRK-52E Cells

**Figure 5:**
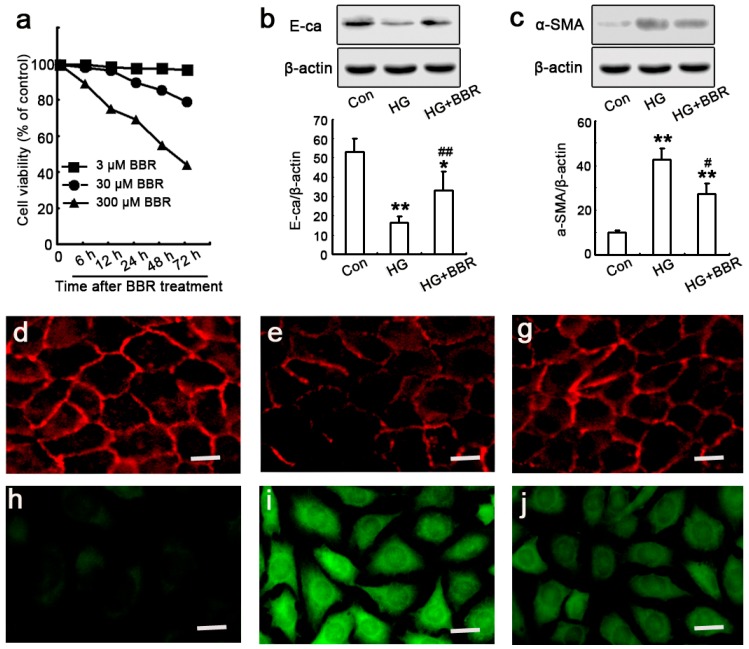
Effects of BBR on HG-induced EMT in the NRK-52E cells. (**a**) The NRK-52E cells were treated with various doses of BBR (3, 30 or 300 μM) for 6, 12, 24, 48 or 72 h, and the cell viability was analyzed by MTT assay. The data were mean ± SEM (*n* = 5); (**b**,**c**) The NRK-52E cells were treated with 30 mM HG for 48 h, in the presence or absence of 30 μM BBR. Representative western blot analysis shows that BBR treatment prevents high glucose (HG)-induced (**b**) E-ca downregulation, as well as (**c**) á-smooth muscle actin (á-SMA) upregulation at 48 h (*n* = 6). Values represent mean ± SEM of three independent experiments performed (** *p* < 0.01 vs. control; * *p* < 0.05 vs. control; ## *p* < 0.01 vs. HG; # *p* < 0.05 vs. HG); (**d**–**j**) Phase contrast microscopy shows that BBR treatment prevents non-epitheliod phenotype acquisition of the NRK-52E cells. Scale bar, 20 μm. For confocal microscopy of immunofluorescence-stained samples, cells were fixed and stained with a primary antibody against E-ca (**d** through **g**), α-SMA (**h** through **j**).Cultures were untreated (**d**,**h**) or exposed to HG (**e**,**i**) or with the addition of BBR (**g**,**j**). Results are representative of two experiments.

To explore whether BBR decreases diabetes-induced tubulointerstitial fibrosis and the potential mechanisms, NRK-52E cells were used to as model in vitro to evaluate the effect of BBR on renal fibrotic [12]. First, we used MTT assay to select optimal concentrations in vitro and 30 μM BBR sulfate was chosen (Figure 5a) as reported previously [3_ENREF_300]. Next, to verify if HG can successfully induce EMT development, the levels of E-ca and α-SMA of NRK-52E cells were detected by western blot and immunofluorescence staining after cultured in HG conditions with or without BBR pretreatment. E-ca is an epithelial marker and plays an essential role in the maintenance of epithelial integrity and even its loss is the earliest key cellular event after tubular EMT induced by HG and a-SMA. Hence it often acts as a mesenchymal marker in renal fibrosis [34]. As shown in Figure 5b,c, the reduction of E-ca protein expression in HG conditions was accompanied by an increase of α-SMA protein expression, which was close associated with our preliminary experiments, and confirmed that HG promotes EMT development in NRK-52E cells [35]. However, HG-induced EMT was decreased after pre-treating the NRK-52E cells with BBR, these demonstrated that BBR could reduce α-SMA and E-ca expression together. Additionally, the inhibitory effect of BBR on HG-stimulated EMT was further confirmed by immunofluorescence staining that HG treatment in the cells for 48 h leading to NRK-52E cell morphology changed into a fibroblast-like shape along with increased and decreased expression of α-SMA and E-ca respectively (Figure 5d–j), which could be attenuated by treating with 30 μM BBR that was consistent with the results of Western blot. These data demonstrated that BBR reversed HG-stimulated EMT in NRK-52E cells.

### 2.5. BBR can Affect HG-Induced Nrf2/HO-1 Signaling in NRK-52E Cells

**Figure 6:**
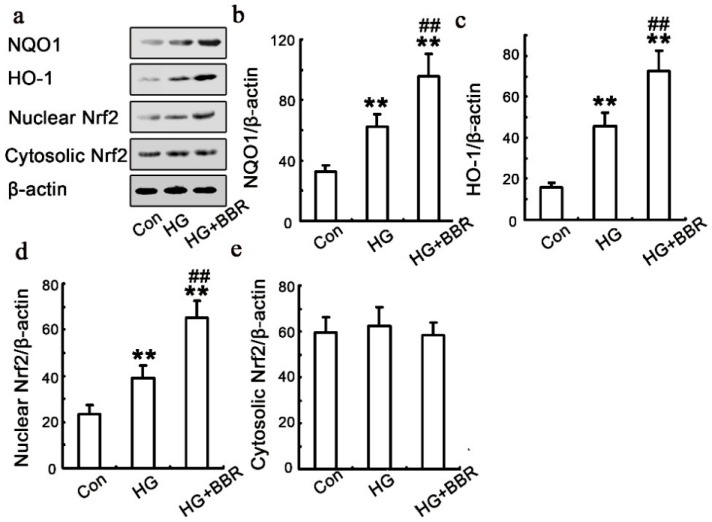
Influence of BBR on HG-induced Nrf2/HO-1 signaling in NRK-52E cells**.** The NRK-52E cells were treated with 30 mM HG for 48 h, in the presence or absence of BBR. The expression NQO1, HO-1 and Nrf2 was analyzed using western blot (*n* = 6). (**a**–**e**) The results were representatives of three independent experiments. β-actin was used as loading control. (** *p* < 0.01 vs. control; ## *p* < 0.01 vs. HG).

HG slightly increased HO-1 and NQO-1expression. BBR treatment with HG enhanced up-regulated HO-1 and NQO-1 expression. In parallel to the effects on the expression of target genes, Nrf2 nuclear levels were increased by Berberine stimulation, while Nrf2 cytoplasmic levels remained relatively even (Figure 6).

### 2.6. Knockdown Nrf2 Abrogates BBR-Induced NQO1 and HO-1 Expression

**Figure 7:**
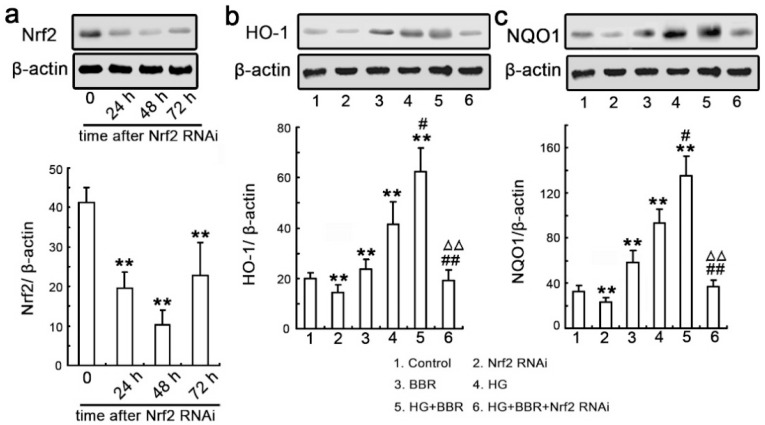
siRNA knockdown of Nrf2 abrogates BBR-induced NQO1 and HO-1 expression. (**a**) NRK-52E cells were transfected with Nrf2-siRNA and western blot analysis was performed with an antibody against Nrf2 was performed at various time-points following transfection (24, 48 and 72 h). Relative Nrf2 expression levels were calculated and normalized to the loading control. Corresponding protein levels were assessed using densitometry and expressed in relative intensities. All results were obtained from three independent experiments. Values are expressed as the mean ± SEM (*n* = 6; ** *p* < 0.01, vs. control); (**b**,**c**) The cells were divided into six groups as description in this paper. Western blotting was performed with an antibody against NQO1 and HO-1. Relative NQO1 and HO-1 expression levels were calculated and normalized to the loading control. Corresponding protein levels were assessed using densitometry and were expressed in relative intensities. All results were obtained from three independent experiments. Values are expressed as the mean ± SEM (*n* = 6) (** *p* < 0.01 vs. control; ## *p* < 0.01 vs. HG; # *p* < 0.05 vs. HG; ΔΔ *p* < 0.01 vs. HG + BBR).

Next, we further examined the impact of Nrf2 on NQO1 and HO-1 expressions. We used Nrf2-siRNA to identify the Nrf2 real function. Relative expression of Nrf2 were detected by Western blot at various time-points after siRNA-Nrf2 transfection though transient methods (Figure 7a). Similar to the effects of BBR, the results confirmed that siRNA-Nrf2 significantly inhibited the NQO1 and HO-1 expression (Figure 7b,c).

### 2.7. The Antifibrosis Effect of BBR on DN Is Depended on the Inhibition of Nrf2 Mediated the TGF-β/Smad Signaling Pathway

**Figure 8:**
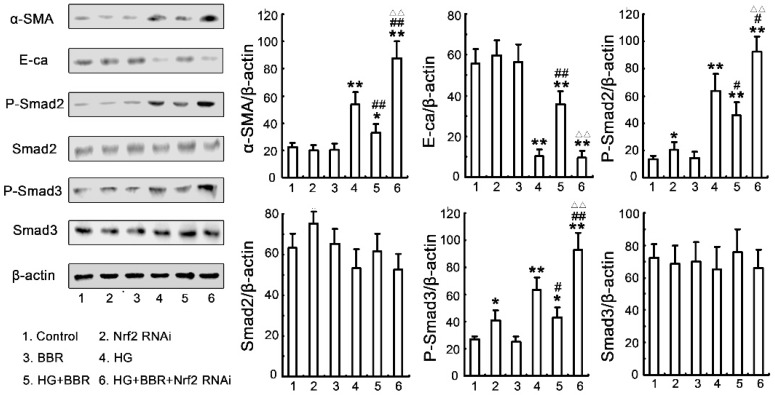
Nrf2 is involved in the inhibitory effect of BBR on the TGF-β/Smad signaling pathway. NRK-52E cells were divided into six groups as mentioned above, corresponding protein levels of Smad2/3, phospho-Smad2/3 and EMT markers were assessed using densitometry and expressed in relative intensities. All results were obtained from three independent experiments. All results were obtained from three independent experiments. Values are expressed as the mean ± SEM (*n* = 6) (* *p* < 0.05 vs. control; ** *p* < 0.01 vs. control; ## *p*< 0.01 vs. HG; # *p* < 0.05 vs. HG; ΔΔ *p*< 0.01 vs. HG + BBR).

It has been proofed that the TGF-β/Smad pathway plays an important role in the pathogenesis of ECM accumulation in diabetic nephropathy. Therefore, we used the Nrf2 siRNA technology to determine if BBR-mediated protection against the HG-induced EMT by activating of antioxidant factor-Nrf2 and inhibition of Smad2/3. As shown in Figure 8, the level of phospho-Smad2/3 of in HG-treated NRK-52E cells was significantly increased comparison with those in control group. In contrast, the level of phospho-Smad2/3 in HG-induced NRK-52E pretreated with 30 um BBR was dramatically decreased. Similarly, the inhibitory effect of BBR on the activity of TGF-/Smad signaling pathway and the increases of phospho-Smad2/3 level was canceled by siRNA-Nrf2. Furthermore, the level of EMT marker proteins in HG-treated cells was significantly increased and the Nrf2-siRNA reversed the inhibitory the protective effects of BBR on HG-stimulated EMT maker protein expression. These results were consistent with the notion that BBR-induced anti-fibrosis effects depend on activating Nrf2-mediated TGF-β/Smad inhibition to protect the NRK-52E cells from HG-induced EMT processes.

## 3. Discussion

In the present study, we demonstrated that BBR treatment not only markedly ameliorated renal dysfunction, but also significantly increased the Nrf2 expression and attenuated the progression of early stage tubulointerstitial fibrosis in STZ-induced diabetic mice. Moreover, the study of mechanism in BBR prevented HG-induced EMT events is because BBR can abrogate HG-induced oxidative stress and induce the expression of Nrf2 and two Nrf2-targeted anti-oxidative genes NQO-1 and HO-1 in NRK-52E cells. More importantly, knockdown of Nrf2 with small interfering RNA abolished the BBR-induced NQO-1 and HO-1 expression. Knockdown of Nrf2 canceled the inhibitory effect of BBR on HG-induced TGF-β/Smad signaling activation. These findings demonstrated that BBR ameliorate the renal injury in diabetic mice by activating Nrf2 and inhibiting TGF-β/Smad pathway.

In DN, EMT of mature tubular epithelial cells of the kidney has been considered involving in the progression of tubulointerstitial fibrosis by accumulation of renal accumulation of matrix proteins [36,37]. Hyperglycemia induced EMT of tubular cells was usually views as the initial factor, which can result to matrix accumulation and deposition, so EMT is also a key mechanism of renal tubulointerstitial fibrosis in DN. Hence, targeting EMT has been as a potential therapeutic method to attenuate the progression of renal fibrogenesis in the diabetic kidney. Tubular EMT is also defined as epithelial cell loss epithelial cell-cell adhesion owing to the down-regulation of E-ca, which can induce TGF β1 activation under the HG conditions and lead to the accumulation of interstitial fibroblasts and decline in renal excretory function [38]. In addition, tubular EMT can produce many ECM components and further to assemble in the extracellular compartment, these can cause massive tissue fibrosis development as seen in diseased kidney. Previous studies indicated that BBR could ameliorate liver fibrosis by suppressing hepatic oxidative stress, fibrogenic potential, and lipid peroxidation [39,40]. Another study also demonstrated the beneficial effects of BBR against bleomycin mediated fibrotic challenge through activating Nrf2 and suppressing NF-κB dependent inflammatory and TGF-β1 mediated fibrotic events in pulmonary [41]. Although glomerulosclerosis is a defined feature of DN and tubulointerstitial injury, which can that determine the rate of decline in renal function [31,42,43], little is known about the role and underlying mechanism of BBR in tubulointerstitial, especially in DN. In the present study, it has been confirmed that HG can induce the changes of EMT markers through decreasing the epithelial marker E-ca and increasing α-SMA than that in the control. Moreover, BBR pretreatment may protect against HG-induced EMT and decrease the α-SMA and E-ca expression, which were associated with the EMT in NRK-52E cells.

Patients with DN usually are caused by EMT in the tubular epithelial cells and these things are usually regarded to be the result of hyperglycemia-induced oxidative stress, but this symptoms of EMT events in tubular epithelial cells can be reverse by anti-oxidants effect [11,12,37]. Nrf2, as a transcription factor, is one critical regulator of anti-oxidant response and an essential signaling factor, has been found that it can protect in many animal disease models, including oxidative stress caused lung injury, fibrosis, asthma and brain ischemia-reperfusion [44,45]. Recent studies have supported the potential therapy of Nrf2 in diabetes, which controlled oxidative stress and regulated inflammatory cytokines [46,47]. Moreover, the protective role of Nrf2 against renal damage, mediating free radicals, was demonstrated by STZ-induced diabetic models [14,47]. In contrast, the levels of nitric oxide metabolites in urine were found increased in Nrf2 knockout mice with STZ-induced diabetes so aggravating renal injury [48]. These data demonstrated that up-regulation of Nrf2 is a potential therapeutic target by mitigating oxidative stress-induced tissue injury. On mechanism, a study also reveals that BBR activates Nrf2 nuclear translocation and protects against oxidative damage via a PI3K/Akt-dependent mechanism in NSC34 motor neuron-like cells, which leads to be increased Nrf2 binding to the antioxidant response element in the promoters of target genes [49]. Most importantly, BBR has been demonstrated that it can attenuate hyperglycemia-induced apoptotic death and promote Nrf2-dependent NGF protein expression and neurite outgrowth, providing a potential therapeutic use of BBR on the treatment of diabetic complication [27]. In the present study, an animal model was used to demonstrate that BBR significantly decreased diabetes-induced renal oxidative damage and tubulointerstitial fibrosis in vivo, which connects with the upregulation of Nrf2 expression. Renal Nrf2 expression remained slight increase in diabetic mice at 12 weeks after diabetes onset, these results may be related to the time of treatment was relatively short. For mechanism, Nrf2 is verified that it can quickly be up-regulated in cells and tissues after exposed to various oxidative stresses while down-regulated in cells or tissue after exposed to chronic oxidative stress [50]. Interestingly, we also found when cells were exposured to high glucose, BBR can enhance the translocation of Nrf2 into the nucleus and upregulated Nrf2-driven antioxidant systems effectively so that attenuates cellular oxidative stress under diabetic conditions.

Previous studies have indicated that upregulation of the NQO1 and HO-1 genes can be widely used for assessment of Nrf2 signaling activation [51]. The production of NQO1 and HO-1 expression by BBR has been verified in several type cell lines including human renal tubular cells and renal fibroblasts. A previous study demonstrates that BBR treatment may be related with AMPK pathway together with Nrf2 pathway may lead to the increase of NQO1 and HO-1 in both LPS-shocked macrophages and mice, and that means AMPK is a upstream of Nrf2 [52]. In addition, BBR can induce the activation of PI3K/Akt and p38 pathway, which both can upregulate the expression and activity of HO-1 as well as NQO1 [35]. Moreover, other studies have revealed that BBR as an Nrf2 activator against glucose neurotoxicity so that attenuating high glucose-induced neurotoxicity, these findings provided another potential therapeutic use of BBR on the treatment of diabetic complications [27]. A previous study has suggested that the NRF2-HO-1system plays a protective role against CsA-induced renal fibrosis by changing EMT gene [15]. In addition, HO-1 deficiency proofed that HO-1 was associated with the increase of fibrosis, tubular TGF-β1 expression, inflammation, and EMT process in renal fibrosis [53]. However, a few known natural substances stimulate NQO1 and HO-1 expression in tubulointerstitial fibrosis in DN. In the study, we further discover the possible downstream mechanisms how Nrf2 affect the protection of BBR from tubulointerstitial fibrosis in DN, the expression of NQO1 and HO-1 was investigated in NRK-52E cells after treated with BBR and HG following Nrf2 siRNA. Consistently, our data presently demonstrated that Nrf2 activation is essential for BBR-stimulated NQO1 and HO-1 expression based on the results of Nrf2-siRNA which inhibited BBR-induced NQO1 and HO-1 protein expression. Simultaneously, the role of HG suppressed E-ca reduction and upregulated α-SMA was reversed by BBR, whereas knockdown of Nrf2 with siRNA down-regulated the anti-fibrosis effect of BBR. These findings suggest that the protection of BBR is depended on the Nrf2 related pathway so that they can active subsequently the key target genes to protect NRK-52E cells from HG-induced EMT processes.

As known that TGF-βis usually high expressed in variety of renal disease, including obstructive nephropathy, and TGF-β, as a major mediator of ECM, can induce the fibrosis development in diabetic nephropathy and tubulointerstitial fibrosis by phoshorylating downstream fators Smad2/3 which can induce the expression of ECM proteins such as fibronectin and collagen-I [54,55,56]. This phenomenon has been confirmed in diabetic animal models and diabetic patients [57,58,59]. In contrast, chronic treatment of *db/db* mice with TGF-β-neutralizing antibodies markedly diminished the expression of collagen and fibronectin and reduced mesangial matrix expansion [2,60]. Therefore, suppressing the activation of TGF-β signaling has been supposed a good therapeutic approach for preventing renal fibrosis [38,55]. Additionally, a previous study demonstrated that sulforaphane can effectively induce Nrf2 activation and inhibited hepatic fibrosis via suppressing TGF-β/Smad signaling [21]. Recent study suggested that DMF can attenuated renal fibrosis by activating Nrf2 and inhibiting ARE-independent TGF-β/Smad signaling pathway. These studies have confirmed that BBR inhibited HG-induced EMT through the Nrf2 and TGF/Smad signaling pathways and it also suggest that increase of oxidative stress and TGF/Smad activation have been linked to the development and progression of diabetic complications including diabetic nephropathy.

## 4. Materials and Methods

### 4.1. Animal Treatment

Six-eight weeks aged C57BL/6J mice (20~25 g of bodyweight) were purchased from Experimental Animal Center of China Medical University (Shenyang, China). All animal experiments were approved by the Experimental Animal Ethical Committee of China Medical University and performed according with the NIH Guide for the Care and Use of Laboratory Animals. Firstly, these mice were randomly divided into three groups, (1) Normal group; (2) diabetic group; (3) BBR-treated group. For streptozotocin (STZ)-induced diabetic mice model, the mice were injected with STZ 150 mg/kg (Sigma, St. Louis, MO, USA) diluted in 0.1 M citrate buffer (pH 4.5) by intraperitoneal injection as described previously [34], when the blood glucose levels of STZ-induce diabetic mice were >16 mmol/L, these mice were diagnosed as diabetic. For BBR-treated diabetic group, these mice were treated with oral BBR (200 mg/kg) in distilled water every day. Mice in the Normal and the diabetic groups were respectively administered orally with equal volume of distilled water. For 12 weeks after diabetic model establishment (at the age of 21–23 weeks), the mice were moved to metabolic cages. At the last day, their urine albumin was collected and detected with Murine ELISA kit (Exocell, Inc., Philadelphia, PA, USA). Finally, the mice were sacrificed, and their blood samples and right kidneys were collected, and tissues samples were fixed in 10% buffered formalin for immunofluorescence staining.

### 4.2. Cell Culture

The NRK 52E normal rat kidney tubular epithelial cells were purchased from the American Type Culture Collection (Manassas, VA, USA). The cells were maintained in DMEM (low glucose; Gibco Life Technologies, Grand Island, NY, USA) with 10% FBS (Gibco), 4 mM L glutamine (Boster Biological Technology Ltd., Wuhan, China) and 1% penicillin/streptomycin (Sigma Aldrich, St. Louis, MO, USA) in a humidified atmoshoere containing 5% CO_2_. Before drugs treatment, NRK 52E cells were cultured in serum free DMEM for 24 h at 37 °C to arrest and synchronize cell growth. In vitro, the cells were divided into six groups, (1) Control group, in which cells were treated with fresh serum free DMEM with 5 mM glucose; (2) siRNA group, cells were transfected with Nrf2 siRNA; (3) BBR group, cells were given with 30 μM BBR for 24 h; (4) HG group, cells were treated with 30 mM HG for 48 h; (5) HG/BBR group, in which cells were treated with with 30 mM HG at 37 °C (Boster Biological Technology Ltd.) for 48 h after pretreatment with 30 μM BBR 24 h; (6) HG/BBR/Nrf2 siRNA group, in which cells, after transfected with Nrf2 siRNA 24 h, were treated with 30 μM BBR for 24 h and next following with 30 mM HG for 48 h. Finally, the concentration of glucose in these cells were detected as previously described 36.

### 4.3. Cell Viability

The cellular viability was detected with MTT assay. Briefly, 10 μL MTT (500 μg/mL; Sigma, St. Louis, MO, USA) was added to 100 µL culture medium in 96-well plates. After incubated for 3 h at 37 °C, MTT solution was removed and 100 μL DMSO (Sigma, St. Louis, MO, USA) was added to dissolve the formazan crystals. Finally, the absorbance was measured at 540 nm with Sunrise microplate reader (Tecan Group Ltd., Männedorf, Switzerland).

### 4.4. Transient Transfection with Nrf2-Small Interfering RNA (siRNA)

The cells were plated in 6-well plates at a density of 2 × 10^5^ cells/well in 2 mL DMEM. Next day, cells were transfected with Nrf2 specific siRNA (sense, 5′ GCACGGU GGAGUUCAUGATT 3′ and antisense, 5′ UCAUUGAACUCCACCGUGCCT 3′) (Santa Cruz Biotechnology, Inc., San Francisco, CA, USA). According to Lipofectamine^®^ 3000 manufacturer’s instructions (Invitrogen Life Technologies, Carlsbad, CA, USA), the cells were transfected with siRNA when they reached 50%–60% confluence in the plate. The cells were re-incubated in medium with 10% FBS after transfected 24 h. The knockdown effect of Nrf2 in these cells was verified by Western blot after transfected 24 h.

### 4.5. Immunofluorescence Staining

The expression of lectin, collagen-I and α-SMA (α-smooth muscle actin) in mice kidneys were detected in the cryostat sections. Firstly, these cryostat sections of mice kidneys were incubated with normal donkey serum (1:20) or anti-lectin (1:100) (polyclonal antibody, Sigma) or anti-collagen I (1:100) or anti-α-SMA (1:100) for 1 h or overnight at RT. Then rinsing with PBS, these sections were incubated with DAR-FITC (1:50) and Texas Red-DAM (1:50) for 2 h at RT. Finally, a confocal laser scanning microscope was used to analyze the expression and location of this target protein (CLSM, SP2, Leica, Germany). All antibodies were purchased from Jackson ImmunoResearch (West Grove, PA, USA).

For immunofluorescence staining of E cadherin (E ca) and α SMA in NRK-52E cells, cells were fixed in 4% paraformaldehyde and permeabilized in 0.1% Triton X-100 at first, and then incubated with mouse monoclonal anti-E-ca (1:100) and mouse monoclonal anti-α-SMA (1:100) as described above. Finally, the cells were incubated with DAR-FITC (1:50) and Texas Red-DAM (1:50) for 2 h at RT. The fluorescent images were visualized with a Fluoview 300 fluorescence microscope (Olympus, Tokyo, Japan).

### 4.6. Western Blot Analysis

Goat polyclonal anti-α SMA, mouse monoclonal anti-E-ca, mouse monoclonal anti Nrf2, mouse monoclonal anti HO-1, mouse anti-NQO1, mouse anti-Keap1, and mouse anti β-actin were purchased from Santa Cruz Biotechnology (San Francisco, CA, USA), while Smad2, Smad3, p-Smad2, p-Smad3 were bought from Cell Signaling Technology (3 Trask Lane Danvers, Danvers, MA, USA). Western blot analysis was performed as previously described [61]. Briefly, equal amounts of protein extracts were loaded to SDS-PAGE 10% gels, and the proteins were transferred to PVDF membranes (Millipore, Temecula, CA, USA). Then the blots were incubated with primary antibodies after blocking with 5% fat-free milk and washed with TBST. Next, the membranes were then incubated with HRP-conjugated secondary antibodies and washed repeated with TBST. After then, the membranes were added with enhanced chemiluminescence kit (Walterson Biotechnology Inc., Beijing, China) and imaged with G BOX EF Chemi HR16 gel imaging system (Syngene, Frederick, MD, USA), Finally, the bands of blots were analyzed and quantified with ChemiDoc™ XRS system (Bio-Rad, Hercules, CA, USA) with Quantity One software version 4.6 (Bio-Rad Laboratories, Inc., Bio-Rad, Hercules, CA, USA). The blots were repeated at least three times for each condition.

### 4.7. Statistical Analysis

Data were displayed with the means ± standard error. All data were analyzed with One-way ANVA and Tukey’s multiple comparison tests. The differences were considered to be significant at *p* < 0.05.

## 5. Conclusions

In summary, the present study provides evidence that BBR inhibits tubulointerstitial fibrosis in DN in vivo and HG induces EMT in NRK-52E cells in vitro by including Nrf2-mediated anti-oxidative effects and suppression of TGF-β/Smad signaling pathway. Therefore, these findings mean BBR can act as a beneficial and potential drug for the prevention or treatment of tubulointerstitial fibrosis in DN.

## Figures and Tables

**Table 1:** Effects of BBR on metabolic and biochemical parameters in STZ-induced diabetic mice.

Items	Control	DM	DM + BBR
Blood glucose (mM)	5.15 ± 0.12	26.32 ± 4.43 **	15.48 ± 4.97 **^,##^
Body weight (g)	29.3 ± 1.15	20.35 ± 2.58 *	24.92 ± 2.16
Kidney weight (g)	0.33 ± 0.03	0.31 ± 0.02	0.32 ± 0.04
KW/BW (%)	1.13 ± 0.02	1.52 ± 0.08 *	1.28 ± 0.05 ^#^
BUN (mM)	6.12 ± 0.53	41.39 ± 5.33 **	19.63 ± 2.87 **^,##^
Cr (µM)	28.54 ± 1.68	87.57 ± 11.61 **	45.82 ± 6.17 **^,##^
Albuminuria (µg/24 h)	9.7 ± 0.58	42.3 ± 3.86 **	25.9 ± 2.17 **^,##^

Results are presented as means ± SEM. (* *p* < 0.05 vs. control group; ** *p* < 0.01 vs. control group; ## *p* < 0.01 vs. control diabetic group; # *p* < 0.05 vs. control diabetic group. *n* = 8).
